# Burstein–Moss-Driven Exciton Dynamics in Degenerately Doped ZnO Quantum Dots

**DOI:** 10.1021/acs.jpcc.6c01256

**Published:** 2026-03-23

**Authors:** Mandeep Singh, Andrea Iudica, Fabio Marangi, Benjamin J. Roman, Delia J. Milliron, Francesco Scotognella, Giuseppe Maria Paternò

**Affiliations:** † IMEM-CNR Institute, Via Alla Cascata 56/C, Povo, Trento 38123, Italy; ‡ Physics Department, 18981 Politecnico di Milano, Piazza L. da Vinci 32, Milano 20133, Italy; § Center for Nano Science and Technology@PoliMi, Istituto Italiano di Tecnologia, Via Raffaele Rubattino, 81, Milano 20134, Italy; ∥ Department of Chemistry, 12330 University of Texas at Austin, 2506 Speedway, Austin, Texas 78712, United States; ⊥ McKetta Department of Chemical Engineering, University of Texas at Austin, 200 E Dean Keeton Street, Austin, Texas 78712, United States; # Department of Applied Science and Technology, 19032 Politecnico di Torino, Corso Duca Degli Abruzzi 24, Torino 10129, Italy

## Abstract

Zinc oxide (ZnO) is a wide-band gap semiconductor with high exciton binding energy and is promising for future optoelectronics. Degenerate doping in ZnO quantum dots (QDs) enables tunable localized surface plasmon resonance (LSPR) in the IR region, but its impact on exciton dynamics remains unexplored. Here, we employ transient absorption spectroscopy to probe exciton dynamics in aluminum-doped ZnO (AZO) QDs. Excitation above the band gap generates hot excitons that relax via thermalization, Auger recombination, and stimulated emission, along with band gap renormalization. The presence of free charge carriers in the conduction band (CB) due to degenerate doping screens Coulomb exciton interactions that leads to suppression of biexciton formation and reduction in Auger recombination lifetime. Despite complete bleaching of ground-state absorption, reminiscent of zero-threshold gain in precharged QDs, no optical gain is achieved in AZO QDs due to doping-induced nonradiative pathways. Our findings highlight how the Burstein–Moss (BM) effect shapes exciton dynamics in degenerately doped wide-band gap QDs.

## Graphical abstract

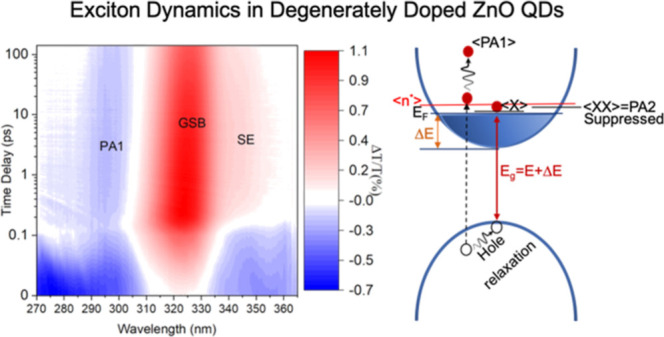

## 1 Introduction

Understanding exciton dynamics at the nanoscale is essential for advancing fundamental optoelectronic and photonic technologies.^1,2^ Quantum dots (QDs), owing to their quantum confinement and strong Coulomb interactions, exhibit discrete electronic states and unique excitonic properties^3–6^ that are highly sensitive to size, composition, and doping.^1,7^ In particular, degenerate doping in which dopant concentrations are high enough to shift the Fermi level into the conduction band can profoundly alter a semiconductor’s electronic and optical behavior. Indeed, such a high doping can lead to emergence of novel light–matter interaction like the discovery of LSPR in degenerately doped semiconductor QDs like metal oxides (MOXs).^8,9^

Particularly, degenerate aluminum-doped zinc oxides (AZOs) have recently emerged as promising candidates for harvesting energy across the broad solar spectrum, i.e., UV–vis–IR.^10^ This arises from its intrinsic UV-range band gap, the observed photogeneration of charge carriers under visible-light irradiation, and tunable LSPR in the infrared region.^8,10^ Moreover, undoped ZnO stands out due to its high exciton binding energy (∼60 meV in bulk) and ability to sustain excitons even at room temperature, making it attractive for energy harvesting and the development of novel optoelectronic devices.^11,12^

While undoped and lightly to moderately doped ZnO has been widely studied,^13–15^ the role of degenerate doping is still not fully understood, especially with respect to the dynamics of exciton formation and relaxation.^16–18^ The Burstein–Moss (BM) effect,^19,20^ where the Fermi level shift blocks transitions from the ground state to the first excited state, forces electrons to occupy higher energy states. This results in a modification of optical and excitonic behavior, particularly in terms of optical absorption,^18^ but the impact on excited-state dynamics remains largely unexplored especially in degenerately doped wide-band gap QDs.

Here, we investigate ultrafast exciton dynamics in degenerately doped AZO QDs by using femtosecond transient absorption spectroscopy (TAS). Using a simple sol–gel synthesis, we achieved controlled synthesis of AZO QDs with up to 40% Al incorporation. Comparison with undoped ZnO QDs reveals several key effects of degenerate doping: exciton decay accelerated by an order of magnitude, suppression of biexciton formation due to Coulomb screening, and altered stimulated emission linked to BM-driven exciton dynamics. We also experimentally tested the Klimov^21^ zero-threshold gain hypothesis, finding that although ground-state absorption is fully bleached and Auger losses are reduced, population inversion is not achieved, indicating a new excitonic decay channel unique to degenerately doped systems.

These findings provide the first direct insight into how Fermi-level modulation governs ultrafast exciton processes in degenerately doped wide-band gap QDs, establishing a foundation for their integration into next-generation optoelectronic and photonic devices.

## 2 Results and Discussion

### 2.1 Morphology and Linear Optical Properties

Figure 1a,b shows STEM images of spherically symmetric ZnO and 40AZO QDs. Using a simple sol–gel approach, we achieved the controlled synthesis of ZnO QDs incorporating up to 40% Al with a narrow size distribution. Unlike prior reports^22^ where such high doping produced larger nanocrystals (5 nm to 30 nm), our QDs fall in the intermediate-to-strong confinement regime (ZnO Bohr exciton radius = 1.8–2.34 nm),^23,24^ thus an ideal platform to probe exciton dynamics. On the other hand, the steady-state optical properties, i.e., absorption and photoluminescence spectra, of ZnO and 40AZO QDs are shown in Figure 1c–f. The detailed explanation is provided in the Supporting Information (Section 2.1). In summary, both absorption (Figure 1c) and emission spectra (Figure 1d) exhibit a blue shift along with band gap widening (Figure 1e) upon Al doping, indicating the fingerprints of the Burstein–Moss effect.^17,18^ As a result of 40% Al doping, the Fermi-level penetration of the conduction band (CB) causes the blocking of the low-energy transition from the VB to the CB.

**Figure 1:**
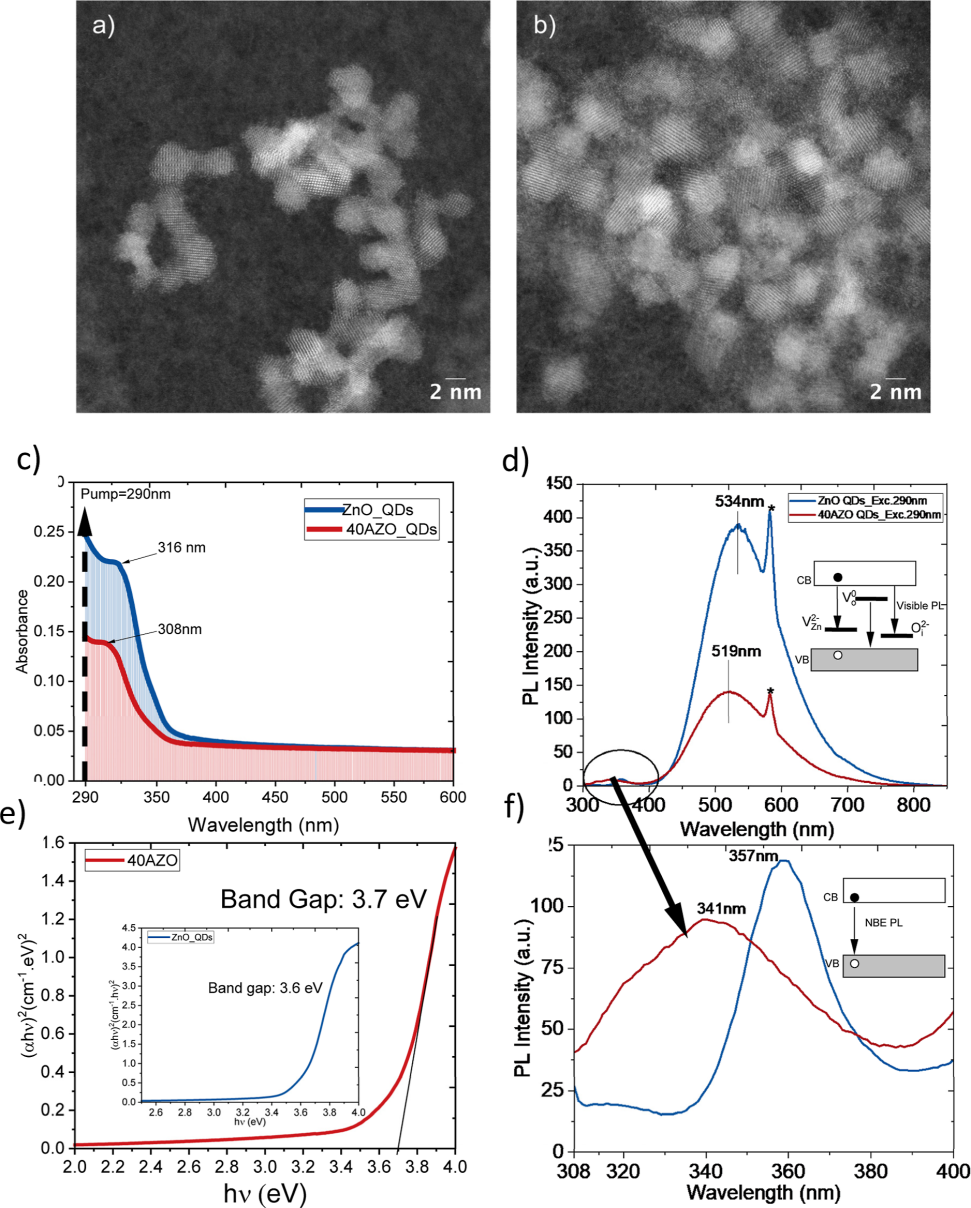
STEM images of spherically symmetric (a) ZnO and (b) 40AZO QDs with an average size of 3.3 ± 0.4 nm and 3.4 ± 0.3 nm, respectively. (a) Linear absorption spectra of ZnO and 40AZO QDS exhibiting a blue shift upon doping indicating the band gap widening. (b) Tauc plots for optical band gap estimation. Clearly after doping, the band gap was found to increase from 3.6 to 3.7 eV. Both blue shift and band gap increase after doping, manifestation of the Burstein–Moss effect. (c) Photoluminescence (PL) spectra showing pronounced visible-range emission, known to originate from the various defects such as singly/doubly ionized oxygen vacancies (VO), interstitial zinc, etc.; (d) near-band-edge (NBE) PL of undoped ZnO and 40AZO QDs, indicating the e-h recombination. Similarly, to the linear absorption, PL peaks also exhibit a blue shift after doping. The pump wavelength (290 nm, blue arrow in Figure 2a) lies closer to the blue-shifted absorption peak of 40AZO QDs than that of pristine ZnO, a resonance difference crucial for understanding their distinct band gap renormalization dynamics. Peaks labeled with “*” in (c,d) are second-order diffraction artifacts from the emission monochromator.

### 2.2 Exciton Dynamics in Degenerately Doped QDs and Their Comparison with Undoped QDs

The differential transmission (Δ*T*/T) spectra at different time-delays and the contour plot for ZnO (a,b) and 40AZO QDs (c,d) are presented in Figure 2. Comparing the dynamics of both QDs, the very first apparent effect is the blue shift in the transient spectra of 40AZO QDs due to the BM effect.^19^ The comparative discussion of all the signals is as follows.

**Figure 2:**
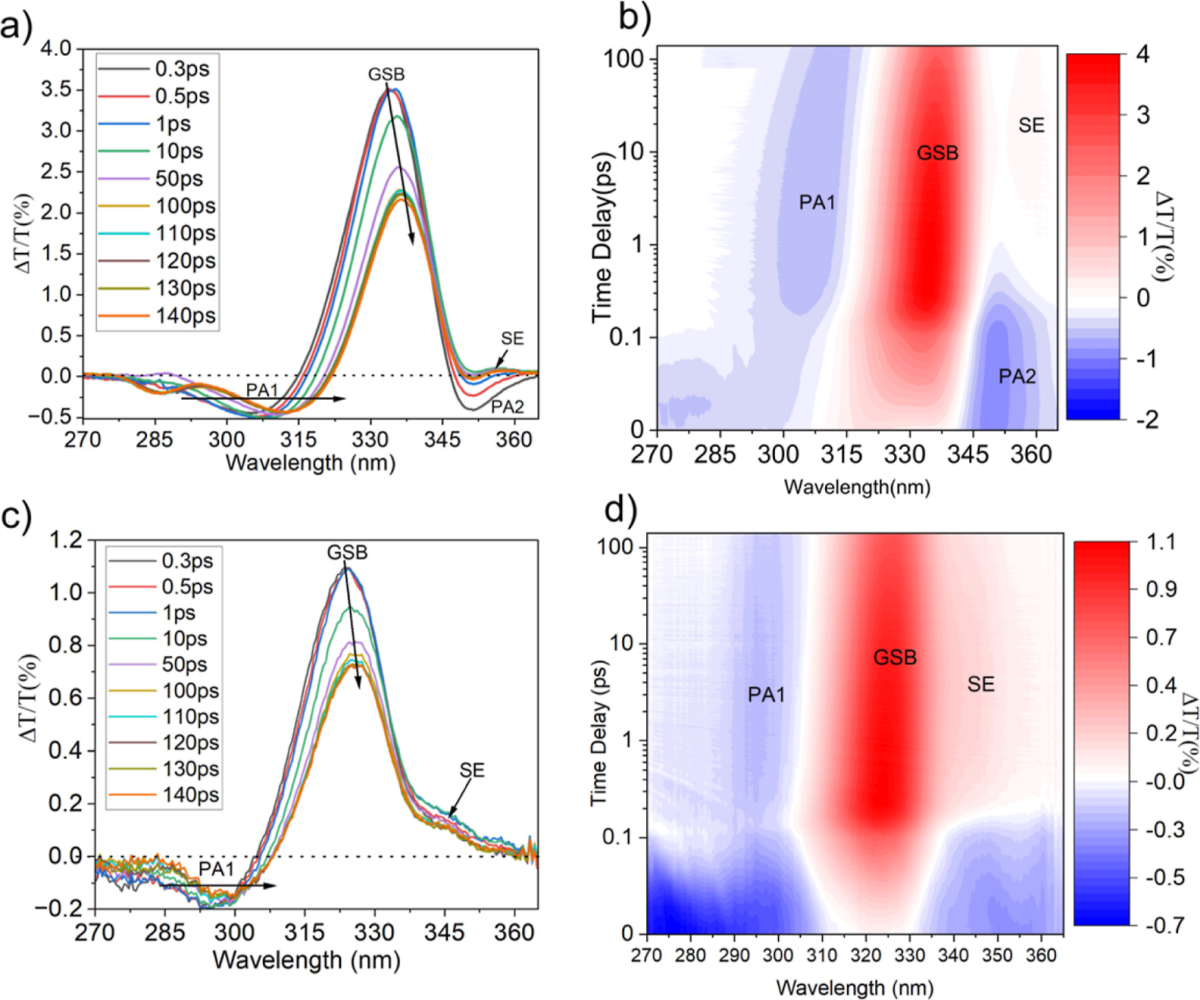
Transient absorption spectra and contour maps. (a) Δ*T*/*T* spectra and (b) contour plot of ZnO QDs; (c) Δ*T*/*T* spectra and (d) contour plot of 40AZO QDs. Both QDs were excited at 290 nm with a fluence of approximately 204 μJ·cm^–2^. However, the red shift is clearly less pronounced in 40AZO QDs due to their blue-shifted absorption edge, creating relatively more resonance condition to the pump wavelength/energy. On the other hand, the absorption edge of undoped-ZnO QDs lies relatively far, hence an enhanced red shift in GSB has been observed.

### 2.3 Hot Excitons and Band Gap Renormalization

The strongest feature in both types of QDs is a positive signal at ∼335 nm (ZnO QDs) and ∼325 nm (40AZO QDs), corresponding to the ground-state bleach (GSB) of the excitonic band-edge transition. This bleaching arises due to phase-space filling in the conduction band and reflects the depletion of available states in the ground state, signaling the formation of electron–hole pairs at the excitonic band edge.^25^ Two clear features are observed in the GSB signal: a red shift and a decrease in intensity with increasing Δ*t*. The time-dependent red shift, indicative of the thermalization and band gap renormalization (BGR).^6^ Particularly, BGR arises as the high energy pump (*E*_pump_ > *E*_g_) generates “hot excitons”^4^ that induce strong carrier–carrier interactions and Coulomb screening, temporarily reducing the band gap.^6^ Consistently, the decrease in intensity over time reflects the underplay of the radiative/nonradiative recombination of excitons.

In the higher-energy (lower-wavelength) region (Figure 2a–d), a negative Δ*T*/*T* signal, labeled as photoinduced absorption (PA1), has been attributed to excited-state absorption (ESA). This PA1 signal originates from the transition of hot excitons to the higher excited state probed by the probe pulse and associated with the BGR.^26^ Notably, similar high-energy PIA features attributed to BGR have also been reported in recent transient absorption studies of semiconductor nanocrystals/QDs.^27–31^ In fact, similar to GSB, PA1 also exhibits a red shift, which suggests that BGR affects not only the band edge but also the entire electronic structure of the QDs. It is worth mentioning that previous studies have also attributed such PA to forbidden exciton transition^32^ in tightly confined perovskite-based QDs due to the existence of band-edge excitons.

### 2.4 Biexciton Formation in Undoped QDs and Their Suppression due to Degenerate Doping

A third key feature is PA2, observed at ∼347 nm, appearing only in the case of ZnO QDs as shown in Figure 2a,b. This feature decays within 1 ps and then transitions into a strong positive signal. The early time dynamics responsible for PA2 are linked to the multiexciton interactions or specifically the biexcitons Stark effect^25,26,29,33–37^ which is well-documented in quantum dots and nanocrystals. Go Yumoto et al.^35^ have attributed biexciton-related PA to the interactions via Coulomb interactions, between hot exciton generated by the pump pulse and band-edge exciton generated by the probe pulse. This attractive exciton–exciton interaction leads to the red shift in band-edge transition. Before the relaxation of hot excitons is completed (0 ps < Δ*t* < 1 ps), the transient red shift of the band edge gives rise to induced absorption and bleaching features around the band edge, resulting in a derivative-like spectral signature. Furthermore, the assignment of PA2 to biexcitons is supported by its narrow and well-defined spectral shape, which distinguishes it from broader PA features associated with continuum or surface states. According to a report by P. Tyagi et al.,^33^ PA due to biexcitons is narrow and discrete, unlike continuum states that span a broad energy range. Furthermore, the fast decay (∼1 ps) of PA2 is consistent with biexciton recombination dynamics or annihilation via the Auger recombination process.^26,32,38^ This process competes with radiative recombination and leads to a fast decay of the biexciton population. Indeed, the transition to the strong positive signal after Δ*t* > 1 ps, attributed to the relaxation of “hot excitons” to band-edge minima, results in bleaching via thermalization, phase-space filling, and Pauli blocking.^26^

In contrast, PA2 is absent in 40AZO QDs due to degenerate doping, which fills low-energy conduction band states with free carriers. These carriers screen the exciton Coulomb interaction, accelerating exciton decay and suppressing biexciton formation. As a result, the longer-wavelength biexciton-induced absorption (PA2) seen in undoped QDs vanishes in degenerately doped ones. This suppression, consistent with Ag­(I)-doped CdSe nanotetrapods,^39^ underscores the Burstein–Moss effect’s pivotal role in shaping excited-state absorption pathways.

### 2.5 Stimulated Emission: Interplay of Biexciton Formation in Undoped QDs and Screening of Exciton Coulomb Interaction in Doped QDs

Finally, the last feature is an additional positive signal observed in both ZnO and 40AZO QDs (see Figure 2a–d), which we assigned to stimulated emission (SE). This claim is supported by (a) the absence of related features in linear absorption spectra; (b) clear probe-delay dependence, distinguishing it from spontaneous emission; and (c) a negative to positive signal transition over time: a hallmark of SE.^40,41^

In ZnO QDs, the SE appears at ∼357 nm, matching the NBE PL of the ZnO QDs (Figure 3d) and peaking at ∼10 ps. This suggests that the emission pathway originates from the recombination of electron–hole pairs at the single excitonic edge. The energy separation between the excitonic GSB and SE estimates the biexciton binding energy which is found to be ∼228 meV.^41^ This value is unusually high due to extreme quantum confinement, which enhances Coulomb interactions in these QDs.^25,26,42,43^ Moreover, SE emerges as negative PA2 disappears, suggesting a direct correlation between biexciton decay and SE signal buildup. The timing of the SE signal (starting at ∼1 ps and peaking at ∼10 ps) coincides with the decay of the biexcitons, suggesting biexciton-mediated SE.

**Figure 3:**
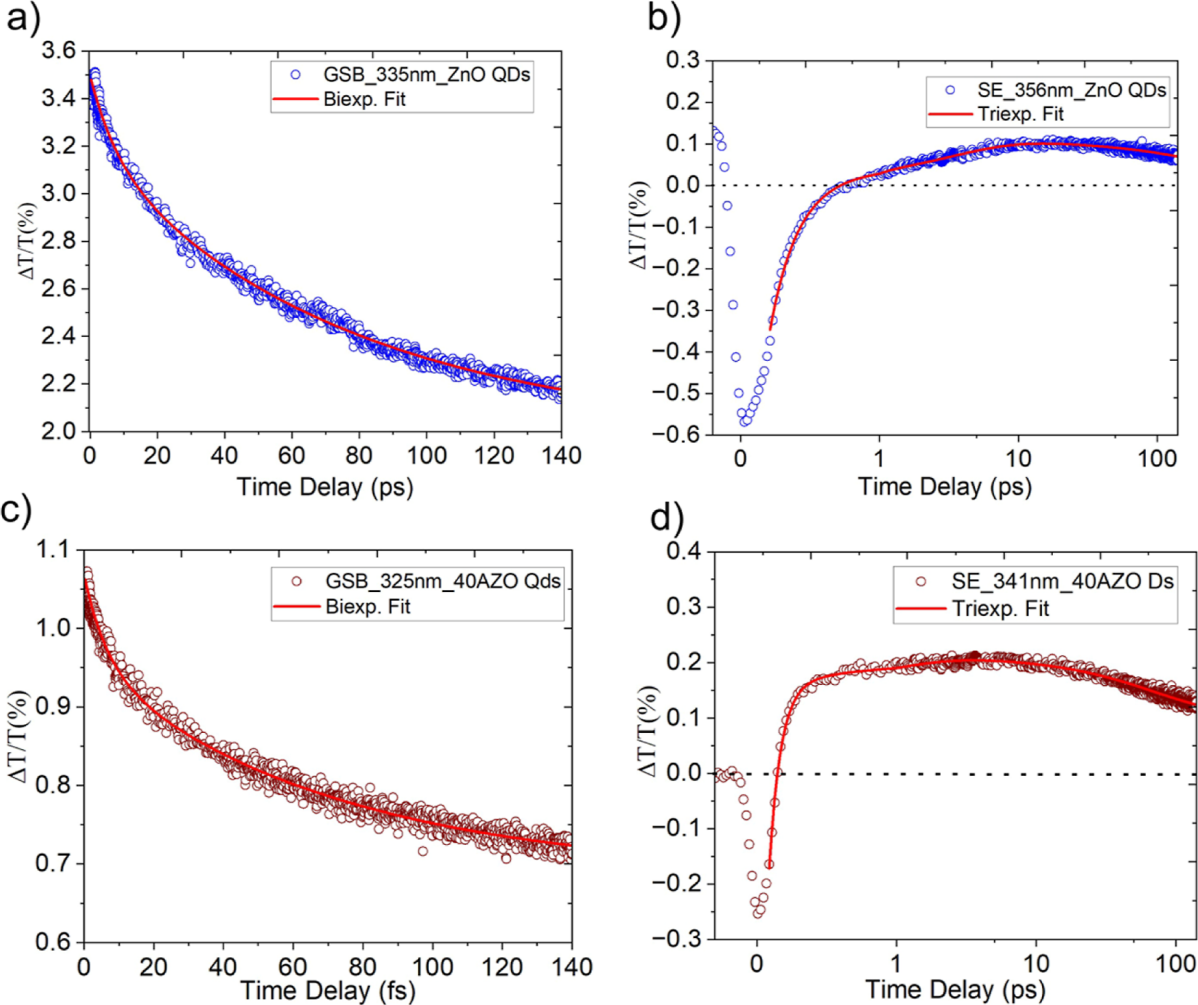
Dynamics of bleach and stimulated emission. (a) GSB dynamics and (b) SE dynamics of ZnO QDs; (c) GSB dynamics and (d) SE dynamics of 40AZO QDs. Excitation was performed at 290 nm with a fluence of approximately 204 μJ·cm^–2^. The data was fitted with the following multiexponential function:^40^ ΔTT=Σ−Αexp(tτi) . The rate of formation of the SE signal in both ZnO and 40AZO QDs is significantly slower compared to the ground-state bleach (peaks at 0.5 ps), with the SE signal reaching its maximum intensity around 10 ps—highlighting the delayed radiative relaxation of excitons relative to their rapid formation after the excitation with the pump.

On the other hand, in 40AZO QDs, SE appears at ∼341 nm (again at the NBE PL), thus confirming its excitonic radiative origin. Unlike ZnO QDs, the SE signal develops almost near time-zero, indicating much faster exciton dynamics. This is due to degenerate doping, which suppresses biexciton formation because of the screening of exciton Coulomb interactions, allowing excitons to decay rapidly without biexciton-induced delay.

### 2.6 Dynamics of Bleach and Stimulated Emission

For a deeper understanding, the dynamics of GBS and SE are presented in Figure 3a,b and Figure 3c,d for ZnO and 40AZO QDs, respectively. The GSB in both ZnO and 40AZO QDs shows an ultrafast rise at near time-zero, indicating hot-exciton^44^ generation, followed by a biexponential decay.^1^ In ZnO QDs, the short component (7.9 ± 0.4 ps) arises from biexciton Auger recombination,^1^ whereas in 40AZO QDs (6.5 ± 0.3 ps), it results from free-carrier-assisted Auger recombination due to degenerate doping. Similarly, P. Bhandari et al.^39^ have observed the significant suppression of Auger recombination in the Ag (I)-doped CdSe nanotetrapods.

While, the longer decay components in both QDs (ZnO QD: 76.6 ± 1.8 ps and 40AZO QDs: 70 ± 2.1 ps) are associated with the radiative decay of excitons, i.e., the near-band-edge (NBE) PL of ZnO and 40AZO QDs. Similar decay features were observed in the cases of pulsed laser deposited ZnO thin films.^44^ The faster decay dynamics in degenerately doped ZnO QDs arises from free carriers in the CB that screen the e–h Coulomb interaction, forcing them to decay faster and suppress the biexciton formation in 40AZO QDs.

On the other hand, the SE band in the case of ZnO QDs can be divided into two distinct regions: (i) a negative ultrafast excited-state absorption event before the Δ*t* < 0.7 ps and (ii) a positive signal corresponding to the SE which appears after 0.7 ps and remains until our measurement time window, which is 120 ps. Similar types of SE dynamics were previously reported in the study of trion emission in CdSe nanoplatelets.^40^ The absorption event before Δ*t* < 0.7 ps reflects the exciton-to-biexcitons transition,^43^ which occurs due to the strong Coulombic interaction between the excitons in a confined quantum space in early time-delay. In contrast, ultrafast buildup of the SE signal in 40AZO QDs is observed near time-zero with no indication of any prior absorption event and is more intense than the ZnO QDs. This is again due to the presence of free charge carriers in the CB of 40AZO QDs, which screens the exciton Coulomb interaction and leads to the suppression of the biexciton-PA event. Hence, the rapid radiative decay of excitons occurs in the form of NBE PL of 40AZO QDs. Furthermore, the dynamics of SE in ZnO quantum dots (QDs) is triexponential, with the time constants of 0.2 ps, 3.2 ps, and >100 ps corresponding to different physical processes. The 0.2 ps component is primarily attributed to the thermalization of hot excitons.^26^ The 3.2 ps and >100 ps decay components are again attributed to biexciton Auger recombination^45^ and radiative recombination of single excitons.^44^

On the other hand, the SE in 40AZO QDs exhibits 10-times faster dynamics than undoped QDs, with 0.08 ps (ultrafast thermalization),^26^ 0.9 ps (free-carrier-assisted Auger recombination^39^), and 69 ps (radiative recombination of excitons^44^) decays. These faster time constants as compared to undoped ZnO QDs demonstrate the critical role of Al doping in modifying exciton dynamics and enhancing carrier scattering, leading to quicker recombination and faster decay times compared to those of undoped ZnO QDs.

### 2.7 Fluence Dependence Dynamics of Degenerately Doped ZnO QDs

V. Klimov et al.^21^ demonstrated that precharging CdS QDs with 1–2 electrons bleach ground-state absorption, enabling “zero-threshold optical gain”. While this effect was shown for lightly charged QDs, the influence of heavy doping remains unclear. In 40AZO QDs, degenerate doping suppresses biexciton-induced PA and fully bleaches the ground-state absorption, suggesting a possible optical gain. To determine this, we investigate the fluence dependence of SE (Figure 4a), normalized to the GSB. At the lowest fluence (13 μJ/cm^2^), a broad spectral shoulder appears alongside the GSB, suggesting an emerging SE contribution. As the fluence increased to 25 μJ/cm^2^, the SE intensity increases and the peak becomes narrower, indicating a more efficient population of band-edge states. Additionally, at 25 μJ/cm^2^, the SE exhibits a blue shift, likely due to state filling effects and Burstein–Moss shift,^46^ indicating an increase in the effective band gap. A similar increase in the intrinsic band gap with pump fluence was observed in the CH_3_NH_3_PbI_3_ thin film.^46^ At pump fluences of 64–242 μJ/cm^2^, the SE peak broadens and decreases in intensity. This behavior is attributed to enhanced carrier–carrier scattering and thermal carrier redistribution at high fluences,^47–49^ both of which disrupt the population inversion required for efficient stimulated emission. Notably, no optical gain was observed at 25 μJ/cm^2^, as total absorption (α), i.e., linear absorption + Δ*A*,^21^ did not reveal a negative feature as shown in Figure 4b (similar trends were observed at other fluence values). This suggests that, despite strong SE, the QDs do not achieve full population inversion, likely due to free-carrier effects and nonradiative pathways introduced by doping. The fluence dependence dynamics of GSB and SE for AZO QDs (Figure S2) along with extracted time constants (Table S1) are provided in the SI.

**Figure 4:**
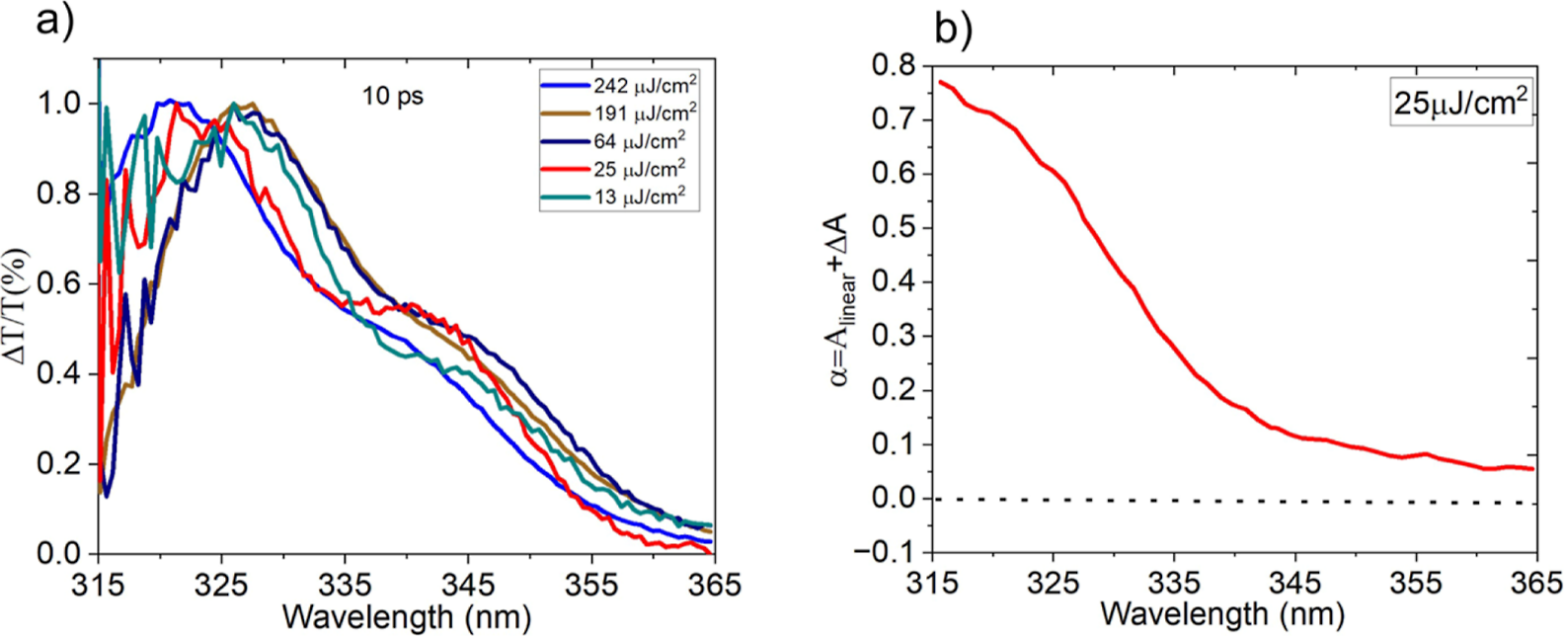
Fluence-dependent ΔT/T spectra (a), GSB dynamics (b), and SE dynamics of 40AZO QDs. An efficient enhancement in the stimulated emission (SE) shoulder is observed at a fluence of 25 μJ/cm^2^ (a). However, optical gain is not achieved (b), primarily due to the presence of free carriers in the conduction band, which introduce nonradiative recombination pathways in 40AZO QDs.

## 3 Discussion

Two major observations can be drawn from the above discussion:

1. Biexciton formation in undoped ZnO QDs.
2. Suppression of biexciton due to the interplay of Burstein–Moss effect and exciton Coulomb-interaction screening due to the presence of free charges in the CB of doped ZnO QDs.

For a qualitative discussion, Figure 5 illustrates the light–matter interaction mechanism in undoped ZnO and 40AZO QDs. In both cases, pumping with energy above the band gap (290 nm > *E*_g_) creates hot excitons ⟨n*⟩ above the CB minimum, depleting the ground state.^50^ The probe pulse then promotes these hot excitons to higher excited states, producing the PA1 signal.^50^ While the PA2 in undoped ZnO QDs indicates the biexciton effect <*XX*>^25^ due to the strong Coulomb attraction between excitons. Further, this PA2 decays within 1 ps to single exciton state <*X*> via Auger recombination and the SE signal appears at the same wavelength as the NBE PL of ZnO QDs, i.e., in the UV region. This signifies e–h recombination that give rises to emission at the single exciton level and system relaxation to the <*n*> state.

**Figure 5:**
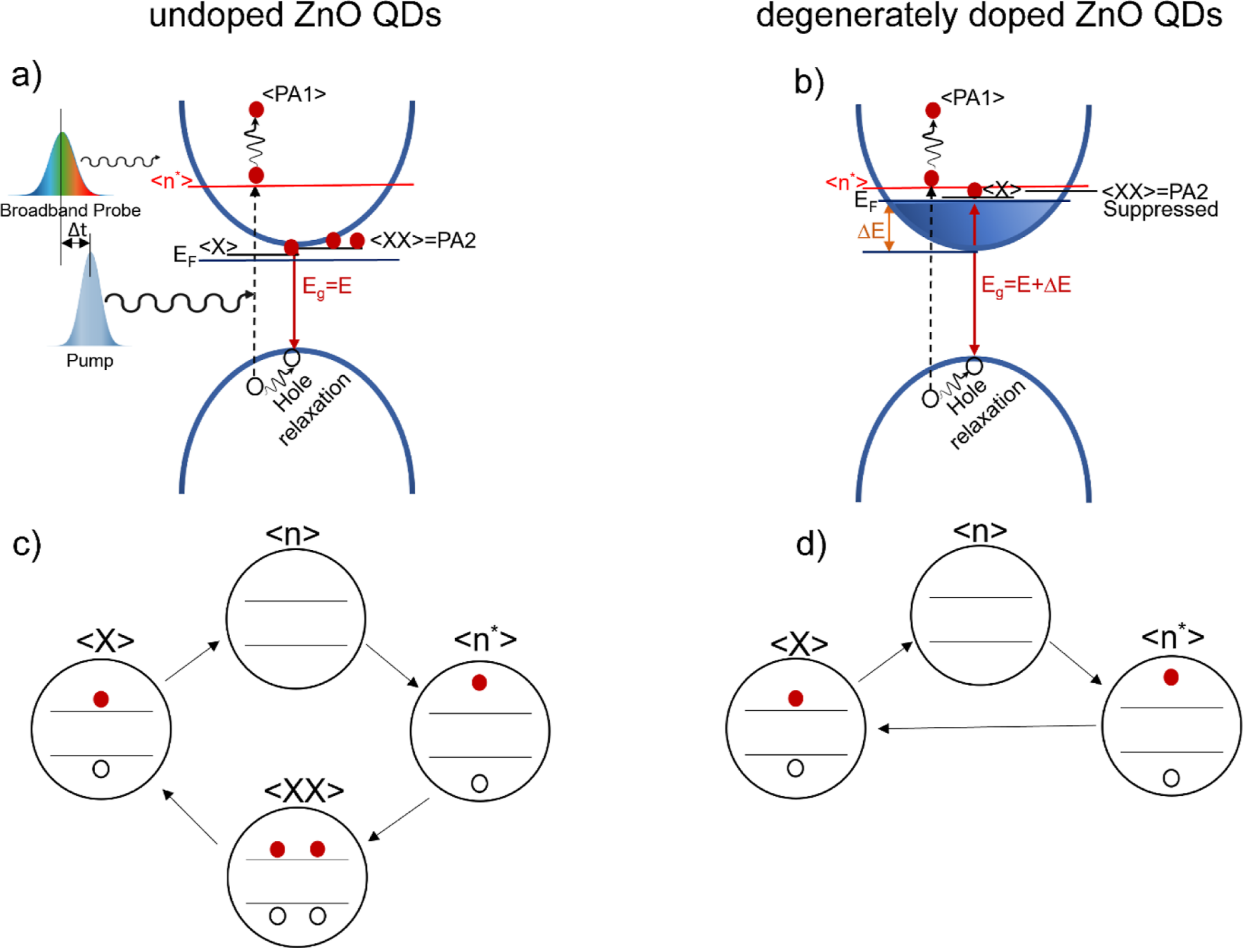
Exciton dynamics in ZnO (a,c) and 40AZO (b,d) QDs. Degenerate doping in 40AZO QDs causes the Fermi level to penetrate into the conduction band, partially filling it with free charge carriers (b). These carriers screen the exciton Coulomb interaction, leading to faster exciton decay and suppression of biexciton formation.

In contrast, 40AZO QDs exhibits SE signal near time-zero alongside a strong GSB due to the suppression of biexciton (no prior absorption even like ZnO QDs). Hence, in 40AZO QDs, hot excitons <n*> immediately decay via free-carrier-assisted Auger recombination to the <*X*> state and then radiatively recombine at the NBE PL of 40AZO QDs leading to the relaxation of 40AZO QDs to the <*n*> state.

## 4 Conclusion

In summary, we investigated exciton dynamics in degenerately doped ZnO quantum dots (QDs) (∼3.4 ± 0.3 nm) by using femtosecond transient absorption spectroscopy. Degenerate doping with 40 atom % Al induces a Burstein–Moss shift, increasing the optical band gap from 3.6 to 3.7 eV, confirmed by absorption spectra and Tauc analysis. Ultrafast excitation at 290 nm generates hot exciton relaxation via thermalization, band gap renormalization (BGR), and Auger recombination. In both undoped and doped QDs, a time-delay-dependent red shift in ground-state bleach (GSB) and photoinduced absorption (PA1) is observed—characteristic of BGR. The shift is less pronounced in doped QDs due to their blue-shifted absorption edge and better resonance with the pump energy, reducing excess carrier temperature. Biexciton formation (∼228 meV binding energy) occurs in undoped QDs, but it is suppressed in doped QDs by Coulomb screening from free carriers. Fluence-dependent measurements on doped QDs reveal stimulated emission (SE) across all pump fluences, peaking at 25 μJ cm^–2^. The SE aligns with near-band-edge photoluminescence (∼341 nm), indicating a fast radiative recombination. Despite full ground-state bleaching, net optical gain is not achieved, likely due to ultrafast nonradiative processes and limited exciton buildup from free carriers.

Our findings highlight how Fermi level penetration and the Burstein–Moss effect reshape exciton dynamics in degenerately doped ZnO QDs, offering insights for ultrafast optical devices based on wide band gap nanomaterials.

## Supplementary Material



## Notes

Experimental section (synthesis of quantum dots and their characterization) and results on linear optical properties of undoped and doped ZnO QDs and fluence dependence dynamics of ground-state bleach and stimulated emission (PDF)
