# Supplementary material for: Burstein–Moss-Driven Exciton Dynamics in Degenerately Doped ZnO Quantum Dots
Source: J Phys Chem C Nanomater Interfaces. 2026 Mar 23;130(13):4885–93. doi: 10.1021/acs.jpcc.6c01256 (PMC13051443; doi:10.1021/acs.jpcc.6c01256)
Supplement: Supplementary file 1 [file jp6c01256_si_001.pdf]

Supporting Information

Burstein–Moss–Driven Exciton Dynamics in Degenerately Doped ZnO Quantum Dot

Mandeep Singh[1,2], *Andrea Iudica*[2], *Fabio Marangi*[3], *Benjamin J. Roman*[4,5], *Delia J. Milliron*[4,5], *Francesco Scotognella*[6] *and Giuseppe Maria Paterno*[2,3]

[1]IMEM-CNR Institute, Via alla Cascata 56/C, Povo-38123-Trento, Italy

[2]Physics Department, Politecnico di Milano, Piazza L. da Vinci 32, 20133 Milano, Italy

[3]Center for Nano Science and Technology@PoliMi, Istituto Italiano di Tecnologia, Via Raffaele Rubattino, 81, 20134 Milano, Italy

[4] Department of Chemistry, University of Texas at Austin, 2506 Speedway, Austin, TX 78712, USA.

[5] McKetta Department of Chemical Engineering, University of Texas at Austin, 200 E Dean Keeton St, Austin, TX 78712, USA

[6]Department of Applied Science and Technology, Politecnico di Torino, Corso Duca degli Abruzzi 24, Torino 10129, Italy

1. Experimental Section

1.1. Synthesis of ZnO QDs using Sol-Gel Chemistry.

To synthesize undoped and Al-doped ZnO QDs, sol-gel chemistry was used.[1] In a typical synthesis of ZnO QDs, sol A was prepared by dissolving 2.28mM zinc acetate dehydrate (Sigma Aldrich) in 10ml ethanol at 70 °C via magnetic stirring. On the other hand, sol B of precipitating agent was prepared by dissolving 9.14mM KOH in 10ml ethanol at room temperature via ultrasonication. After 90 minutes, when complete dissolution of both solutions is achieved, sol B is mixed dropwise into sol A at 70 °C under stirring over a period of 10 minutes. The stirring was continued for another 90 minutes to complete the reaction to finally yield ZnO QDs. Similarly, to synthesize Al-doped ZnO QDs, aluminium nitrate (40at%, Sigma Aldrich) was dissolved together with zinc acetate dehydrate in 10ml ethanol to prepare sol A (2.28mM). The rest of the process is exactly the same as the synthesis of pristine QDs. The undoped QDs were named as ZnO, while Al doped QDs were named as 40AZO.

1.2. **STEM Imaging.**

The ZnO QDs were drop-cast onto Cu TEM grids and imaged using an aberration corrected JEOL NEOARM STEM in dark field with an accelerating voltage of 200 kV.

1.3. **Linear Absorption and PL spectra.**

Absorption spectra in the range 290-600nm were acquired using Perkin Elmer Lambda 1050 spectrophotometer, equipped with deuterium (180-320 nm) and tungsten (320- 3300 nm) lamps and three detectors (photomultiplier 180-860 nm, InGaAs 860-1300 nm and PbS 1300-3300 nm). The absorption spectra were corrected for the reference spectra taken at 100% transmission (without the sample) at 0% transmission (with an internal attenuator).

While PL spectra was acquired in the range 300-900nm with the excitation at 290nm with Cary Eclipse Fluorescence spectrophotometer equipped with a Xenon flash lamp (80 Hz).

1.4. **Pump-probe experimental set-up.**

The main laser source of the TAS measurements is a Ti:Sapphire regenerative amplifier, emitting 100 fs pulses at 800 nm with a repetition rate of 1 kHz. The laser source is separated into two beams, for the pump and probe of the experiment. The UV pump pulses are obtained in two stages:[2,3] Firstly, a noncollinear optical parametric amplifier (NOPA), coupled to a chirped mirror pair compressor, is used to generate broadband (80-100nm FWHM) visible pulses with tunable central wavelength (from 490 to 620 nm), and a short pulse duration of 10 fs. The second harmonic of this pulse is then used to get a UV pulse, with tunable wavelength from 250 nm to 300 nm. For the second harmonic, we employ a thin (0.05 mm) BBO cut at 42°. The UV pulse is then filtered with dichroic mirrors and

recompressed by means of a prism pair compressor. A schematic view of the setup is provided in (Fig. S1). The near UV- visible probe (320-700 nm) is a broadband supercontinuum obtained by focusing the fundamental of the laser on a 2 mm thick CaF_2 plate. We also generate a UV (250-365 nm) supercontinuum by focusing the second harmonic of the fundamental on the same CaF_2 plate. During the experiment, the pump beam photoexcites the sample, while the probe light is used to collect the transmission spectrum of the sample in the photoexcited state, at variable time-delays (Δt). The probe transmitted through the sample is dispersed with a grating and detected with a CCD, where the differential transmission ΔT/Toff ($\Delta T = T_{on} - T_{off}$) is measured, where T_{on} and T_{off} represent the intensities of the transmitted probe in presence and absence of the pump, respectively. An optical chopper, operating at a frequency of 0.5 kHz, allows the shot-to-shot acquisition of the $\Delta T/T_{off}$. The TAS maps are recorded by varying the Δt between pump and probe with an automated delay stage that can reach up to 100ps.

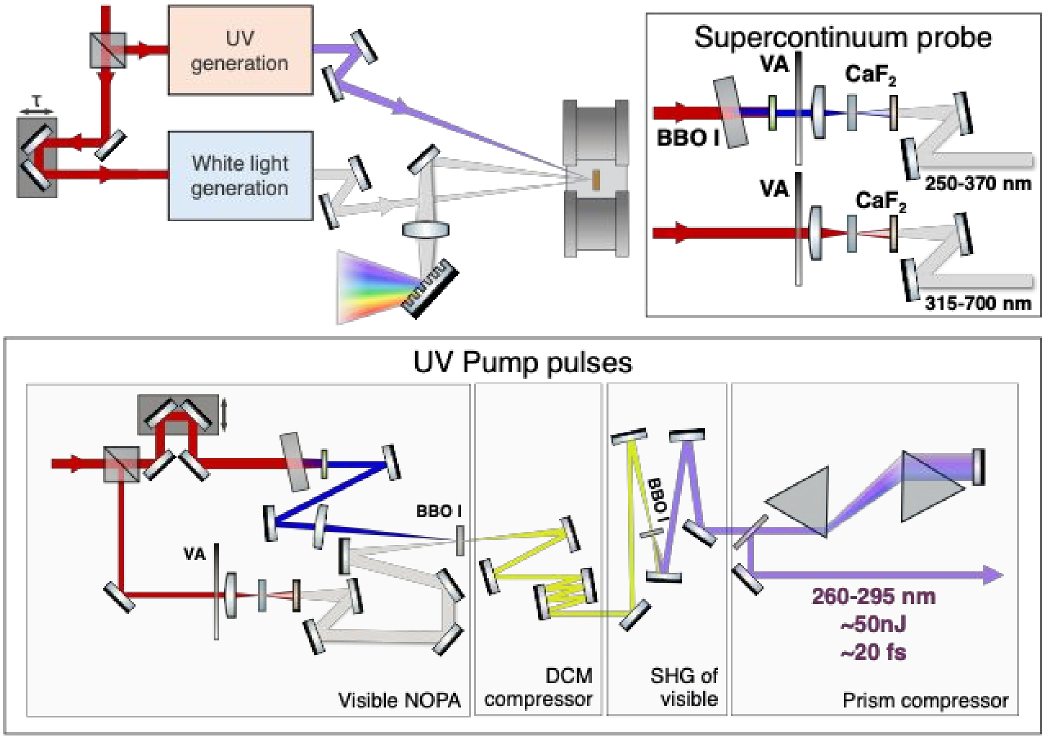

Figure S1: Transient absorption spectroscopy set-up for exciton dynamics analysis.

2. Results

2.1 Linear optical properties of ZnO and 40AZO QDs

The ground state absorption spectra of ZnO and 40AZO QDs dispersed in ethanol are shown in Fig. 2a, exhibit distinct excitonic features.[4] Al doping in ZnO QDs causes a blue shift in the absorption peak from 316 nm (pristine) to 308 nm (40AZO), indicating changes in the electronic structure due to higher carrier concentration.[4,5] The direct band gap energies, determined from Tauc plots ((αhν)2 vs. hν, Fig. 2b), show an increase from 3.6 eV for ZnO QDs to 3.7 eV for 40AZO QDs. This band

gap widening and blue-shift in absorption of 40AZO QDs is a manifestation of the Burstein-Moss effect.[5,6] Al doping donates free electrons that fill lower CB states, pushing the Fermi level into the CB and inducing degeneracy in 40AZO QDs.[6] As a result, the optical transitions from the valence band (VB) to these now-occupied CB states are blocked, forcing transitions to higher energy states, thus causing blue shift of absorption edge. Later, we will see that this degeneracy reduces available phase space for exciton recombination, affecting both radiative and non-radiative dynamics.

The PL spectra of ZnO and 40AZO QDs via excitation at 290 nm exhibit two prominent peaks as shown in figure (Fig. 2c, d): one in the visible region associated with the several different types of defect such as singly/doubly ionized oxygen vacancies (VO), interstitial zinc etc.[7] The second PL peak was observed in the UV region corresponding to near-band-edge (NBE) exciton recombination.[7] In the case of ZnO QDs, the visible peak is centered around 534nm while the UV peak exhibits highest intensity around 357nm. On the other hand, two significant changes were observed upon Al doping (40AZO QDs). First, both peaks shift to higher energies (blue shift), a result of the Burstein-Moss effect where free carriers donated by Al fill the lower conduction band states, effectively widening the optical band gap. Second, the visible defect-related emission at 519 nm is significantly quenched (lower in intensity), likely due to a reduction in recombination through defect states as free electrons in the conduction band screen the defect potential, weakening carrier trapping and radiative recombination. The UV NBE peak at 341nm also experiences slight intensity reduction but broadens due to enhanced carrier scattering, including electron-electron and electron-phonon interactions, arising from the higher free carrier concentration in doped QDs.

In addition to the broad visible PL peaks from both ZnO QDs and 4AZO QDs, sharp peaks (marked with "*") were consistently observed at approximately twice the excitation wavelength (e.g., ~580 nm for 290 nm excitation). Unlike the near-band-edge (NBE) and visible PL, these sharp peaks do not exhibit a blue shift upon degenerate doping. Similar sharp features were also observed when the PL was recorded under excitation at other wavelengths (300 nm, 310 nm, and 320 nm). For 310 nm and 320 nm excitations, additional sharp peaks appeared at the excitation wavelength itself. These features are attributed to second-order diffraction artifacts of the emission monochromator and do not represent intrinsic emission from the QDs. Similar second-order diffraction effects have been reported in fluorescence measurements when higher-order diffraction is not adequately filtered out.

2.2. Fluence Dependence Dynamics of Bleach and Stimulated Emission in 40 AZO QDs

We are again recalling that the white-light probe (315–650 nm) was used in the fluence dependence measurements as our focus is on the stimulated emission (SE) signal at 341 nm and also interested if any contribution from defect related PL of 40AZO QDs at 519 nm (see Fig. 2c) might also observed. As no signal was observed around 519 nm in the TA measurements, the data from 315 nm to 365 nm

was presented. The ground-sate bleach (GSB) and SE emission dynamics showed significant dependence on pump fluence (see fig. S2 (a,b)). Table 1 presents the time constants extracted by fitting the transient absorption dynamics using Equation 1 (see main manuscript). It should be noted that because our delay range is limited to 80 ps, the values of time constants τ_2 (GSB) and τ_3 (SE) were not reported in the table as these components were not fully resolved. We therefore report it as $\tau_{\_slow} > 80$ ps. The short-time constants τ_1 for both GSB and SE which is related to thermalization,[8] showed no significant fluence dependence. While, the τ_2 (SE) which is associated to the free-carrier-assisted Auger recombination,[9] were found to increase as fluence decreases, indicating slower Augur recombination rate. This increase in the decay-constants at lower fluences is attributed to the reduction in the multi-carrier interaction,[10–12] leading to a transition into a single-exciton like regime. In this regime, both radiative and non-radiative decay processes like augur recombination, emission etc. occur at slower rates compared to the multicarrier interaction-dominated regime at higher fluences.

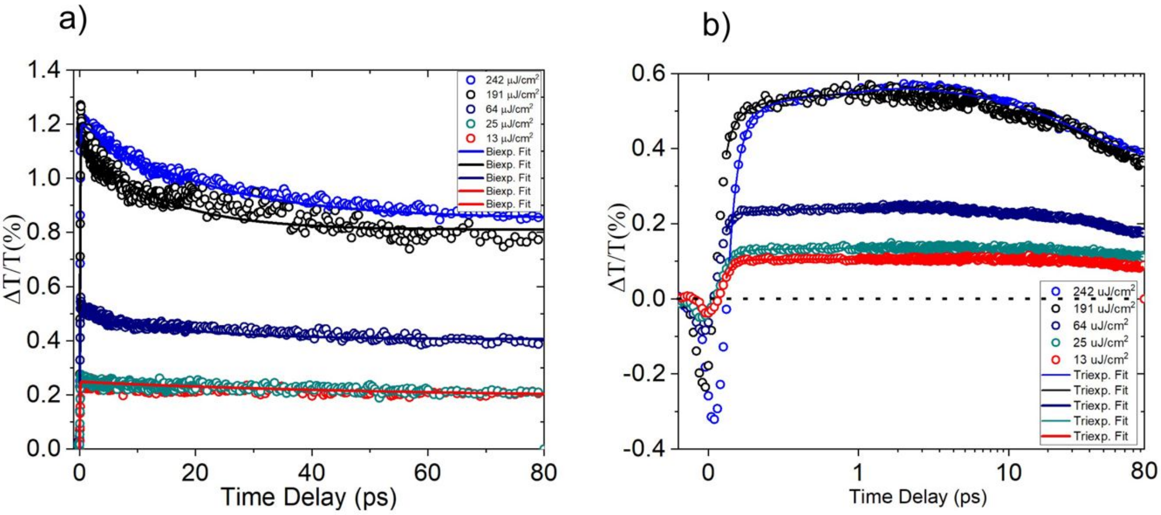

Figure S2: Fluence dependent GSB dynamics (a) and SE dynamics (b) of 40AZO QDs.

**Fluence**	**Ground State Bleach**		**Stimulated Emission**		
(μJ/cm²)	τ_1(ps)	τ_2(ps)	τ_1(ps)	τ_2(ps)	τ_3(ps)
242	0.8±0.03	$\tau_{\_slow} > 80$ ps	0.6±0.02	5.6±0.4	$\tau_{\_slow} > 80$ ps
191	0.7±0.04	$\tau_{\_slow} > 80$ ps	0.4±0.2	2.3±0.5	$\tau_{\_slow} > 80$ ps
64	0.6±0.03	$\tau_{\_slow} > 80$ ps	0.3±0.1	4.7±0.7	$\tau_{\_slow} > 80$ ps
25	0.8±0.04	$\tau_{\_slow} > 80$ ps	0.5±0.1	7.1±2.1	$\tau_{\_slow} > 80$ ps
13	0.6±0.04	$\tau_{\_slow} > 80$ ps	0.4±0.04	36.3±11.5	$\tau_{\_slow} > 80$ ps

Table S1: Fluence dependence of time constants obtained via fitting GSB and SE dynamics of 40AZO QDs

References

(1) Sowik, J.; Miodyńska, M.; Bajorowicz, B.; Mikolajczyk, A.; Lisowski, W.; Klimczuk, T.; Kaczor, D.; Zaleska Medynska, A.; Malankowska, A. Optical and Photocatalytic Properties of Rare Earth Metal-Modified ZnO Quantum Dots. *Applied Surface Science* **2019**, *464*, 651–663. https://doi.org/10.1016/j.apsusc.2018.09.104.

(2) Varillas, R. B.; Candeo, A.; Viola, D.; Garavelli, M.; Silvestri, S. D.; Cerullo, G.; Manzoni, C. Microjoule-Level, Tunable Sub-10 Fs UV Pulses by Broadband Sum-Frequency Generation. *Opt. Lett.* **2014**, *39* (13), 3849–3852. https://doi.org/10.1364/OL.39.003849.

(3) Borrego-Varillas, R.; Ganzer, L.; Cerullo, G.; Manzoni, C. Ultraviolet Transient Absorption Spectrometer with Sub-20-Fs Time Resolution. *Applied Sciences* **2018**, *8* (6). https://doi.org/10.3390/app8060989.

(4) Alexandrov, A.; Zvaigzne, M.; Lypenko, D.; Nabiev, I.; Samokhvalov, P. Al-, Ga-, Mg-, or Li-Doped Zinc Oxide Nanoparticles as Electron Transport Layers for Quantum Dot Light-Emitting Diodes. *Scientific Reports* **2020**, *10* (1), 7496. https://doi.org/10.1038/s41598-020-64263-2.

(5) Raja, M.; Muthukumarasamy, N.; Velauthapillai, D.; Balasundraprabhu, R.; Agilan, S.; Senthil, T. S. Quantum Dot Sensitized Aluminium Doped and Copper Doped ZnO Nanostructure Based Solar Cells. *Journal of Materials Science: Materials in Electronics* **2014**, *25* (11), 5035–5040. https://doi.org/10.1007/s10854-014-2268-5.

(6) Srinet, G.; Kumar, R.; Sajal, V. Effects of Aluminium Doping on Structural and Photoluminescence Properties of ZnO Nanoparticles. *Ceramics International* **2014**, *40* (3), 4025–4031. https://doi.org/10.1016/j.ceramint.2013.08.055.

(7) Brahma, S.; Khatei, J.; Sunkara, S.; Lo, K.-Y.; Shivashankar, S. A. Self-Assembled ZnO Nanoparticles on ZnO Microsheet: Ultrafast Synthesis and Tunable Photoluminescence Properties. *Journal of Physics D: Applied Physics* **2015**, *48* (22), 225305. https://doi.org/10.1088/0022-3727/48/22/225305.

(8) Mondal, A.; Aneesh, J.; Kumar Ravi, V.; Sharma, R.; Mir, W. J.; Beard, M. C.; Nag, A.; Adarsh, K. V. Ultrafast Exciton Many-Body Interactions and Hot-Phonon Bottleneck in Colloidal Cesium Lead Halide Perovskite Nanocrystals. *Physical Review B* **2018**, *98* (11), 115418. https://doi.org/10.1103/PhysRevB.98.115418.

(9) Bhandari, P.; Ali, F.; Datta, A. Suppression of Auger Recombination and Promotion of Carrier Diffusion in Ag(I)-Doped CdSe Nanotetrapods. *J. Phys. Chem. C* **2025**, *129* (27), 12420–12428. https://doi.org/10.1021/acs.jpcc.5c02719.

(10) Wheeler, D. A.; Zhang, J. Z. Exciton Dynamics in Semiconductor Nanocrystals. *Advanced Materials* **2013**, *25* (21), 2878–2896. https://doi.org/10.1002/adma.201300362.

(11) Midgett, A. G.; Hillhouse, H. W.; Hughes, B. K.; Nozik, A. J.; Beard, M. C. Flowing versus Static Conditions for Measuring Multiple Exciton Generation in PbSe Quantum Dots. *The Journal of Physical Chemistry C* **2010**, *114* (41), 17486–17500. https://doi.org/10.1021/jp1057786.

(12) McGuire, J. A.; Sykora, M.; Joo, J.; Pietryga, J. M.; Klimov, V. I. Apparent Versus True Carrier Multiplication Yields in Semiconductor Nanocrystals. *Nano Lett.* **2010**, *10* (6), 2049–2057. https://doi.org/10.1021/nl100177c.
